# Near Miss Ruptured Ectopic Pregnancies: A Case Series of Diagnosis With Point-of-Care Ultrasound and an Independent Review of Radiology Images

**DOI:** 10.7759/cureus.87153

**Published:** 2025-07-02

**Authors:** Jonathan Schonert, Joseph Minardi, Nicolas Denne, Justine Pagenhardt, Nicole Dorinzi

**Affiliations:** 1 Emergency Medicine, St. Luke’s Hospital, Chesterfield, USA; 2 Emergency Medicine, West Virginia University, Morgantown, USA; 3 Emergency Medicine, West Virginia University School of Medicine, Morgantown, USA

**Keywords:** case report, ectopic, first trimester, pocus, vaginal bleeding

## Abstract

First-trimester pregnancy complications are common in the emergency department, with ectopic pregnancy being the leading cause of maternal mortality in this period. Emergency physicians play a critical role in early diagnosis and management, with proficiency in point-of-care ultrasound (POCUS) being an essential skill. Even when comprehensive radiology ultrasound is also available, independent image review remains crucial to ensure that subtle findings are not missed in this timely diagnosis.

We present two cases of ruptured ectopic pregnancy initially evaluated in community emergency departments. Both cases involved lower abdominal pain, vaginal bleeding, and positive pregnancy tests. In each instance, transabdominal POCUS performed during initial evaluation identified concerning findings, including adnexal masses with complex echogenicity and pelvic free fluid. Despite comprehensive radiology ultrasound interpretations not confirming ectopic pregnancy, the emergency physician’s reassessment of imaging raised concern for rupture, prompting urgent gynecologic intervention. Surgical exploration confirmed ruptured ectopic pregnancy in both cases, requiring operative management. Both patients had favorable postoperative outcomes.

Ectopic pregnancy remains a high-risk condition necessitating thorough evaluation. These cases highlight the crucial role of POCUS and independent ultrasound interpretation by emergency physicians in preventing diagnostic delays. Recognizing subtle imaging findings, especially in cases where radiology interpretations are inconclusive, is critical for timely intervention. Emergency physicians should maintain a high index of suspicion and collaborate with gynecology and radiology colleagues to optimize patient outcomes.

## Introduction

First-trimester pregnancy problems are common in the emergency department, and ruptured ectopic pregnancies are the leading cause of maternal mortality in the first trimester [1]. Ectopic pregnancies are defined as any pregnancy implanted outside the uterine cavity, with ruptured ectopics leading to life-threatening intraperitoneal bleeding. Emergency physicians are the initial contact for many of these patients [2]. As such, a high degree of clinical mastery is required to assure timely and accurate diagnoses, appropriate management, and the avoidance of diagnostic errors/delays and resultant morbidity/mortality [3]. Point-of-care ultrasound (POCUS) is an invaluable tool to immediately evaluate these patients [4]. A thorough understanding of the sonographic findings in the first trimester, including normal and abnormal findings, is necessary, even when comprehensive radiology ultrasound is available. Some findings may be subtle, and an independent review of all images is paramount and must be correlated with other clinical data to make timely and appropriate management decisions.

## Case presentation

Case 1

A 27-year-old woman presented to a community emergency department with lower abdominal pain, pelvic cramping, and vaginal bleeding for three weeks. Her periods had been irregular, and she was unsure of her last normal period. Her vital signs were normal, but she appeared to be in pain. She had diffuse abdominal tenderness without peritoneal signs. A transabdominal POCUS was performed immediately at the time of history and physical examination, revealing a mildly enlarged uterus with a small hypoechoic endometrial lesion and pelvic free fluid, some of which appeared complex and echogenic, raising concern for blood (see Video 1).

**Video 1:** Case 1: Ruptured Ectopic Pregnancy Transabdominal POCUS reveals an empty uterus, free fluid in the pelvis, and a possible pseudogestational sac. A complex adnexal mass concerning for ectopic pregnancy is visualized deep in the uterus. Transvaginal imaging provides clearer views, confirming the mass with adjacent free fluid, reinforcing the diagnosis of a ruptured ectopic pregnancy POCUS: point-of-care ultrasound

Laboratory tests were ordered, and she was sent for a comprehensive transvaginal ultrasound by radiology, as an endocavitary transducer was not available in the emergency department at this facility. Her quantitative human chorionic gonadotropin (HCG) returned at 1634 mIU/mL. Pelvic examination showed a closed cervical os, scant blood in the vaginal canal, and diffuse pelvic tenderness. Radiology ultrasound was interpreted as follows: “no sonographic evidence of intrapelvic abnormality.” A review of the radiology-obtained images by the emergency physician raised concern for a complex pelvic mass with surrounding mixed echogenicity fluid (see Video 1). These imaging findings, in conjunction with the positive HCG and clinical picture, were suspicious for a ruptured ectopic pregnancy. Gynecology was consulted and agreed to take the patient for diagnostic laparoscopy, where a ruptured ectopic with hemoperitoneum was confirmed. She underwent salpingo-oophorectomy and did well postoperatively.

Case 2

A 31-year-old woman presented with left-sided abdominal pain. She had been experiencing some abdominal and flank pain for more than a month, but the character had changed with increased intensity of pain in the previous few hours. Additionally, on the day of presentation, she had unexpected vaginal bleeding heavier than her menses and a positive home pregnancy test. Her last period was three months prior. A transabdominal POCUS was performed at the time of the initial history and physical examination, revealing no intra-uterine pregnancy, a simple right ovarian cyst, and a complex lesion in the left adnexa with irregular, mixed echogenicity material that was suspicious for clotted blood (as seen in Video 2). HCG returned at 167 mIU/mL.

**Video 2:** Case 2: Ruptured Ectopic Pregnancy Transabdominal POCUS reveals an empty uterus, a simple ovarian cyst, and irregular fluid collections concerning for free fluid. Further imaging of the left adnexa identifies a complex mass with ill-defined borders, not characteristic of bowel or ovarian tissue. Transvaginal ultrasound provides clearer visualization, confirming an empty uterus, adjacent free fluid, and a complex mass highly suggestive of a ruptured ectopic pregnancy POCUS: point-of-care ultrasound

Pelvic examination revealed a closed cervical os and scant blood in the vaginal canal with left adnexal fullness and tenderness. She was sent for a comprehensive transvaginal ultrasound by radiology, as an endocavitary transducer was not available in the emergency department at this facility. This examination was interpreted as inconclusive, specifically that the mass in the left adnexa did “not have the typical appearance of ectopic pregnancy.” An independent emergency physician review of the images raised concern for a ruptured ectopic given the complex left adnexal mass with irregular, ill-defined borders and mixed echogenicity fluid (see Video 2).

Given that gynecologic services were not available at this facility, the difficult decision to transfer the patient was made. She was accepted to an outside facility and taken for diagnostic laparoscopy, where a ruptured ectopic was confirmed, and she underwent left salpingectomy. She did well postoperatively.

## Discussion

As previously stated, first-trimester pregnancy problems are common in the emergency department. With ectopic pregnancy being the leading cause of maternal mortality in the first trimester [5], every available clinical detail must be scrutinized by the emergency physician to assure timely and accurate diagnoses and to avoid potential morbidity/mortality [5,6].

As illustrated by these cases, as well as others, even when comprehensive radiology services are available, a high index of suspicion is required, and independent review and interpretation of all images are necessary to make an accurate and timely diagnosis [7,8]. Reasonable experience in both performing and interpreting POCUS studies may be beneficial in establishing the necessary knowledge base to confidently and competently interpret these scenarios and make critical management decisions for these patients. This skillset is even more critical in lower-resource settings where radiology ultrasound services may not be readily available and the decision to transfer a patient is one not made lightly.

While many first-trimester pregnancy problems are straightforward, more subtle presentations with less well-defined imaging findings require scrutiny, thoughtful consideration, and a willingness to take ownership of the diagnosis to make difficult patient-centered management decisions. Patients with biochemical evidence of pregnancy yet without a demonstrable intra-uterine pregnancy present a common diagnostic challenge. Every clinical clue including the duration and volume of any vaginal bleeding, the menstrual history, the HCG levels, the physical examination findings, and a detailed sonographic interrogation of the entire female pelvis and the abdomen is necessary in these cases.

Ectopic pregnancy is straightforward when there is evidence of a relatively normal pregnancy in the wrong location. More often, however, given that ectopic pregnancies do not develop normally, they appear as nondescript, usually echogenic or complex extra-uterine masses or even interstitial pregnancies [9]. While simple cystic structures are common in the adnexa, any complex or echogenic extra-uterine mass is strongly suggestive of an ectopic pregnancy unless there is definitive evidence to suggest otherwise [10]. Further, any excessive or complex peritoneal fluid suggests a ruptured ectopic, which drastically changes the management from methotrexate to likely emergent surgery [11,12]. One further clue to the presence of subtle blood in the pelvis is masses or other extra-uterine material with mixed echogenicity and/or ill-defined or irregular borders. This class of findings is sometimes seen with blood in varying stages of coagulation. When peritoneal free fluid is seen, it typically provides a sharp outline and definition of adjacent structures. When simple free fluid is seen without sharp delineation of adjacent structures, further interrogation is warranted to evaluate for nearby complex fluid that may represent incompletely coagulated blood. If there are findings that are suspicious for ruptured ectopic pregnancy, consultation with a gynecologist is warranted for the consideration of diagnostic laparoscopy. In some very select cases, very close clinical observation with serial quantitative HCG levels and ultrasound examinations may be appropriate.

We break down the diagnostic considerations, brief overview of the findings, and other notes in Table 1.

**Table 1:** Ultrasound (US) Diagnostic Categories and Management in Early Pregnancy This table summarizes the typical ultrasound findings and recommended approaches for various early pregnancy presentations HCG: human chorionic gonadotropin

Diagnosis	Findings	Notes
Intra-uterine pregnancy	Clear intra-uterine pregnancy, relatively central fundal endometrial location	Caution: heterotopic and interstitial
Pregnancy of unknown location	Complete evaluation of the female pelvis and abdomen without intra-uterine pregnancy but no extra-uterine pregnancy, no complex mass, and no excessive or complex pelvic or peritoneal fluid	Close follow-up with repeat quantitative HCG and US
Ectopic pregnancy without rupture	Ectopic pregnancy without excessive or complex pelvic or peritoneal fluid	Review indications and contraindications for medical therapy
Ruptured ectopic pregnancy without frank hemoperitoneum or shock	Ectopic pregnancy with complex pelvic fluid but without frank hemoperitoneum	Operative management
Ruptured ectopic pregnancy with frank hemoperitoneum ± shock	Ectopic pregnancy with frank hemoperitoneum	Resuscitation plus operative management

## Conclusions

Ectopic pregnancy is an important first-trimester problem that is commonly evaluated in the emergency department. Sometimes, the imaging findings are not overly clear. Emergency physicians must have a mastery of the spectrum of findings and independently review all diagnostic imaging to avoid diagnostic errors/delays with potential resultant morbidity and mortality. Gaining experience along with additional training with POCUS may assist in developing the requisite knowledge base and experience to confidently make these difficult diagnoses. Continued collaboration between emergency physicians, radiologists, and gynecologists is recommended when evaluating these scenarios that may have ambiguous or less clear findings.
